# Ribosomal Protein S29 Regulates Metabolic Insecticide Resistance through Binding and Degradation of CYP6N3

**DOI:** 10.1371/journal.pone.0094611

**Published:** 2014-04-11

**Authors:** Jing Yu, Shengli Hu, Kai Ma, Linchun Sun, Hongxia Hu, Feifei Zou, Qin Guo, Zhentao Lei, Dan Zhou, Yan Sun, Donghui Zhang, Lei Ma, Bo Shen, Changliang Zhu

**Affiliations:** 1 Department of Pathogen Biology, Nanjing Medical University, Nanjing, China; 2 Pediatric Research Center, Nanjing Children's Hospital Affiliated to Nanjing Medical University, Nanjing, China; National Cheng Kung University Medical College, Taiwan

## Abstract

**Background:**

Many diseases are transmitted by mosquitoes, including malaria, dengue fever, yellow fever, filariasis, and West Nile fever. Chemical control plays a major role in managing mosquito-borne diseases. However, excessive and continuous application of insecticides has caused the development of insecticide resistance in many species including mosquito, and this has become the major obstacle to controlling mosquito-borne diseases. Insecticide resistance is the result of complex polygenic inheritance, and the mechanisms are not well understood. Ribosomal protein RPS29 was found to be associated with DM resistance in our previous study. In this study, we aim to further investigate the involvement of RPS29 in deltamethrin resistance.

**Methodology and Principal Findings:**

In this study, tandem affinity purification was used to identify proteins that can interact with RPS29. Among the candidate proteins, CYP6N3, a member of the CYP450 superfamily, was identified, and binding to RPS29 was confirmed *in vitro* and *in vivo* by GST pull-down and immunofluorescence. CCK-8 assay was used to investigate the RPS29-CTP6N3 interaction in relation to DM resistance. CYP6N3 overexpression significantly enhanced DM resistance and insect cell viability, but this was reversed by RPS29 overexpression. Western blot was used to study the mechanism of interaction between RPS29 and CYP6N3. RPS29 increases CYP6N3 protein degradation through the proteasome.

**Conclusions and Significance:**

These observations indicate that CYP6N3, a novel RPS29-interacting partner, could stimulate deltamethrin resistance in mosquito cells and RPS29 overexpression targeted CYP6N3 for proteosomal degradation, abrogating the CYP6N3-associated resistence to deltamethrin. Our findings provide a novel mechanism associated with CYP450s mediated DM resistance.

## Introduction

Many new and re-emerging diseases are transmitted by mosquitoes, including malaria, dengue fever, yellow fever, filariasis, and West Nile fever [1], [2], [3], [4], [5]. Chemical control plays a major role in managing mosquito-borne diseases. Deltamethrin (DM) is a fourth generation synthetic pyrethroid pesticide that kills insects by overstimulation of the nervous system [6], [7]. Due to low toxicity towards mammals and birds, and limited persistence in soil, DM is commonly used to help control the transmission of insect-borne diseases. However, excessive and continuous application of insecticides has caused the development of insecticide resistance in many species including mosquito, and this has become the major obstacle to controlling mosquito-borne diseases [8], some of which are resurgent. In mosquitoes, the mechanisms responsible for insecticide resistance are mainly associated with target site modification and metabolic resistance. The mechanisms of metabolic resistance often occur through enhanced biodegradation of the insecticide, usually via overexpression and/or elevated activity of three major enzyme families; CYP450s, esterases, and glutathione S-transferases. Currently, it has been proved that CYP450 are the primary enzyme family associated with resistance to pyrethroids such as DM. High levels of P450 activity are frequently observed in pyrethroid-resistant mosquitoes in Africa [9], [10], [11]. CYP450 enzymes were significantly correlated to deltamethrin resistance in populations of mosquitoes from southern China [12]. Resistance-associated genes have been identified and include cytochrome P450s (CYP450s), esterases, GST, and acetylcholinesterase [7]. Insecticide resistance operates through complex polygenic inheritance, and the mechanisms are not well understood. Suppression subtractive hybridization (SSH) and combined cDNA microarray analysis was used to identify genes that are differentially expressed in DM-susceptible and DM-resistant strains of *Culex pipiens pallens* [13]. One of these identified genes was ribosomal protein *s29* (*rps29*), which shares over 98% sequence identity with homologs in five other mosquito species, and 77% with the human homolog. The putative amino acid sequence of RPS29 is identical to that of *Aedes albopictus*, *Culex quinquefasciatus* and *Aedes aegypti* [14]. Ribosomal proteins are involved in protein synthesis in all living cells and are highly conserved from yeasts to mammals. RPS29 is a component of the 40S ribosomal subunit. Studies indicated that human RPS29 has extra-ribosomal functions as an apoptosis inducer and RNase [15], [16], but despite this, insect RPS29 remains poorly characterized. In our previous study, it had been identified that RPS29 is involved in DM resistance of mosquito [14].

To further investigate the involvement of RPS29 in DM resistance, tandem affinity purification (TAP) was used to identify proteins capable of binding to RPS29. CYP6N3, a member of the CYP450 superfamily, was identified, and shown to interact with RPS29 *in vitro* and *in vivo*. CYP6N3 overexpression significantly enhanced mosquito cells viability to DM, but this was reversed by RPS29 overexpression. These data suggest that RPS29 increased the proteosomal degradation of CYP6N3, constituting a novel resistance mechanism.

## Materials and Methods

### Cell lines and plasmids

The C6/36 cell line derived from *Aedes Albopictus* was purchased from the ATCC biological resource centre. Cells were cultured in DMEM medium containing 10% FBS and maintained at 28°C in 5% CO_2_. The pIB-V5 vector was purchased from Invitrogen, USA. Full-length GFP and RPS29 were inserted sequentially into the pIB-V5-His vector, full-length CYP6N3 and MYC which were synthesized (Invitrogen, Carlsbad, USA) and inserted sequentially into the pIB-V5-His vector, and full-length CYP6N3 and RFP were inserted sequentially into the pIB-V5-His vector, according to standard procedures. The cDNAs encoding GS and RPS29 were cloned into the pIB-V5-His expression vector, and cDNAs encoding RPS29 and CYP6N3 were cloned into the pGEX-6p-1 expression vector (downstream of the GST sequence) and the PET-32a expression vector (downstream of the His sequence), respectively.

### Transfection

Plasmids were transfected into cells using a FuGENE HD Kit (Promega,Madison, USA) according to the manufacturer's instructions. RPS29 and CYP6N3 siRNAs and the silencer negative control (sncRNA) were commercially available (GenePharma, Shanghai, China). Transfections with the siRNA oligomers were performed using the X-tremeGENE Kit (Roche, Basel, Switzerland) according to the manufacturer's instructions. All materials used in the siRNA transfections were treated with DEPC.

### GST pull-down assay

GST fusion proteins were bound to glutathione sepharose beads (Genscript, Nanjing, China) and incubated with *in vitro* translated protein at 4°C for 2 h with gentle agitation in binding buffer (20 mM Tris-HCl pH 7.4, 150 mM NaCl, 10% glycerol, 1 mM EDTA, 1 mM DTT, 0.2% NP-40). After extensive washing with binding buffer, bound proteins were analyzed by Western blotting.

### Immunofluorescence and confocal laser scanning microscopy

Cells were transfected with pIB-V5-RPS29-GFP and pIB-V5-CYP6N3-MYC constructs and grown on glass cover slips. After 24 h, cells were fixed with 4% paraform, permeabilized with 0.3% NP-40, and blocked with 3% fetal bovine serum for 1 h. Coverslips were incubated at 4°C overnight with a 1∶500 dilution of anti-MYC primary antibody (Abmart, Shanghai, China), washed, incubated with a 1∶500 dilution of rhodamine-conjugated affinipure goat anti-mouse IgG secondary antibody (Bioworld, Nanjing, China) at 37°C for 1 h, and stained with 5 ng/ml DAPI for 5 min to visualize nuclei with a confocal laser scanning microscope (LSM710, CarlZeiss, Germany).

### Cell viability assay

The Cell Counting Kit-8 (Dojindo, Japan) was used to measure DM resistance in C6/36 transfectants under a range of DM treatments. Cells were seeded in 96-well plates and incubated for 24 h at 28°C in 5% CO_2_. 24 h after transfection, cells were treated with 100 µl of DM in DMSO at 0, 10^0.5^, 10^1^, 10^1.5^, 10^2^, and 10^2.5^ mg/l. After a further 24 h, 10 µl CCK-8 solution were added to each well and incubated for 2 h at 28°C, after which the absorbance at 450 nm was measured using a microplate reader. The final DMSO concentration was 0.5% (v/v) in different concentrations of DM. Experiments were repeated in triplicate.

### RNA isolation, reverse transcription, and real-time PCR

Total RNA was extracted using TRIzol (Invitrogen, Carlsbad, USA) according to the manufacturer's instructions. Reverse transcription was performed according to the manufacturer's instructions (Takara, Japan). Real-time PCR was performed in triplicate using the SYBR Green Mastermix on the ABI Prism 7300 Sequence Detection System (Applied Biosystems, Foster City, USA). The relative gene expression level was calculated through Delta-delta Ct method and β-actin was used as the internal control. Experiments were repeated in triplicate.

### Western blotting

Cells were lysed in RIPA buffer (Biyuntian,Shanghai, China) containing the protease inhibitor PMSF. Lysates were separated by 10% SDS-PAGE. Proteins were transferred onto a nitrocellulose membrane and blocked with 5% skimmed milk for 1 h at 37°C. Membranes were incubated at 4°C overnight with primary antibodies (anti-GST, -HIS, -GFP, -MYC, Abmart, Shanghai, China; anti-Tublin, Baworld, Nanjing, China). Following incubation with horseradish peroxidase–conjugated goat anti-mouse and goat anti-rabbit secondary antibodies (Baworld, Nanjing, China) at 37°C for 1 h, proteins were detected by ECL (Thermo, Rockford, USA) according to the manufacturer's instructions.

### Statistical analysis

Data from three independent experiments were averaged. All data were presented as mean ± SD. Statistical analyses were conducted using the Student's *t*-test in the SPSS 13.0 software package. Confidence intervals were *P<0.05, **P<0.01, ***P<0.001.

## Results

### Detection of RPS29 binding proteins using TAP-MS/MS

**Figure 1:**
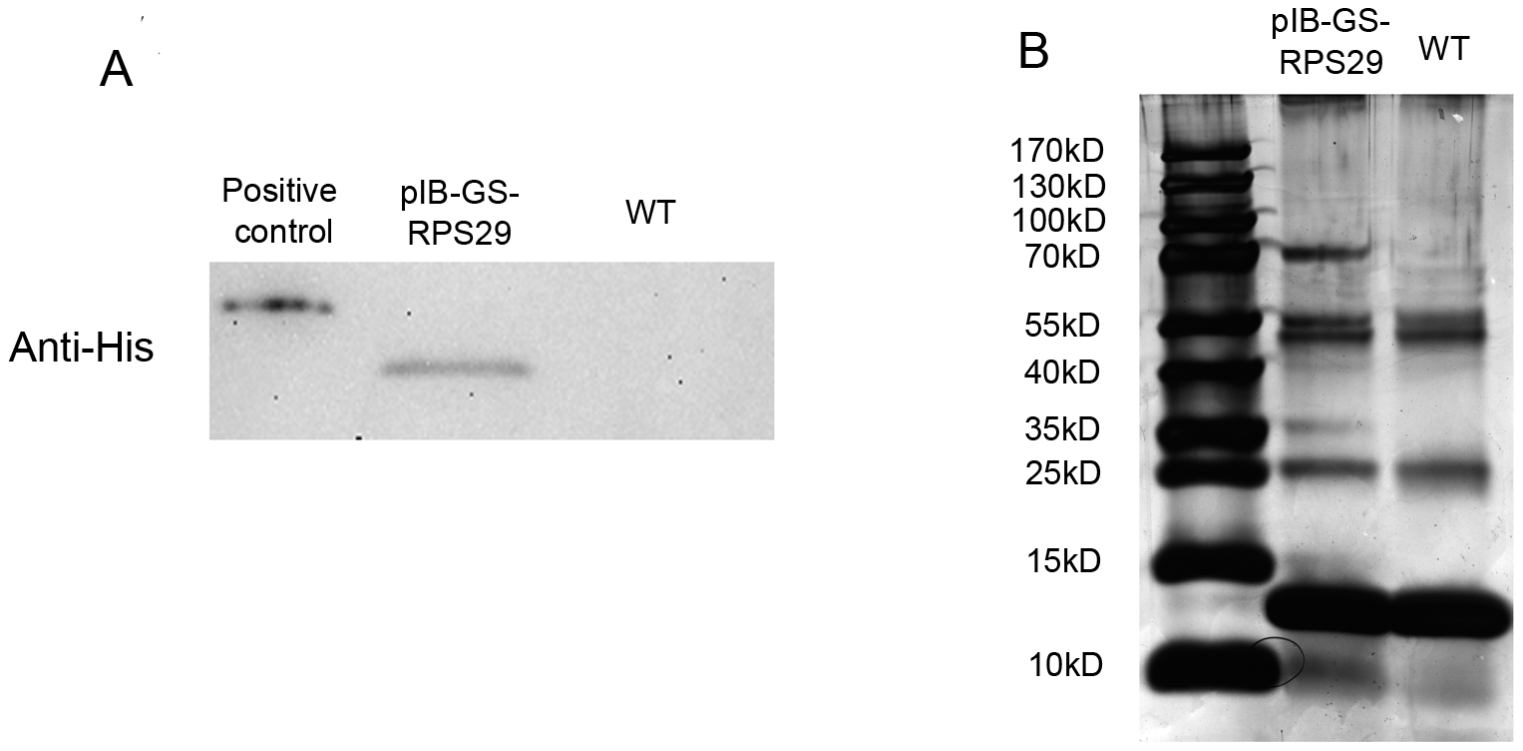
Tandem affinity purification to identify RPS29 interacting proteins. (A) pIB-v5-GS-RPS29 was transfected into C6/36 cells. Constitutive expression of TAP-tagged RPS29 was confirmed by Western blotting using anti-His antibody. (B) SDS-PAGE and silver staining of cell lysates from transfected pIB-v5-GS-RPS29 cell lines. Representative TAP outputs for MS/MS analysis are shown.

**Figure 2:**
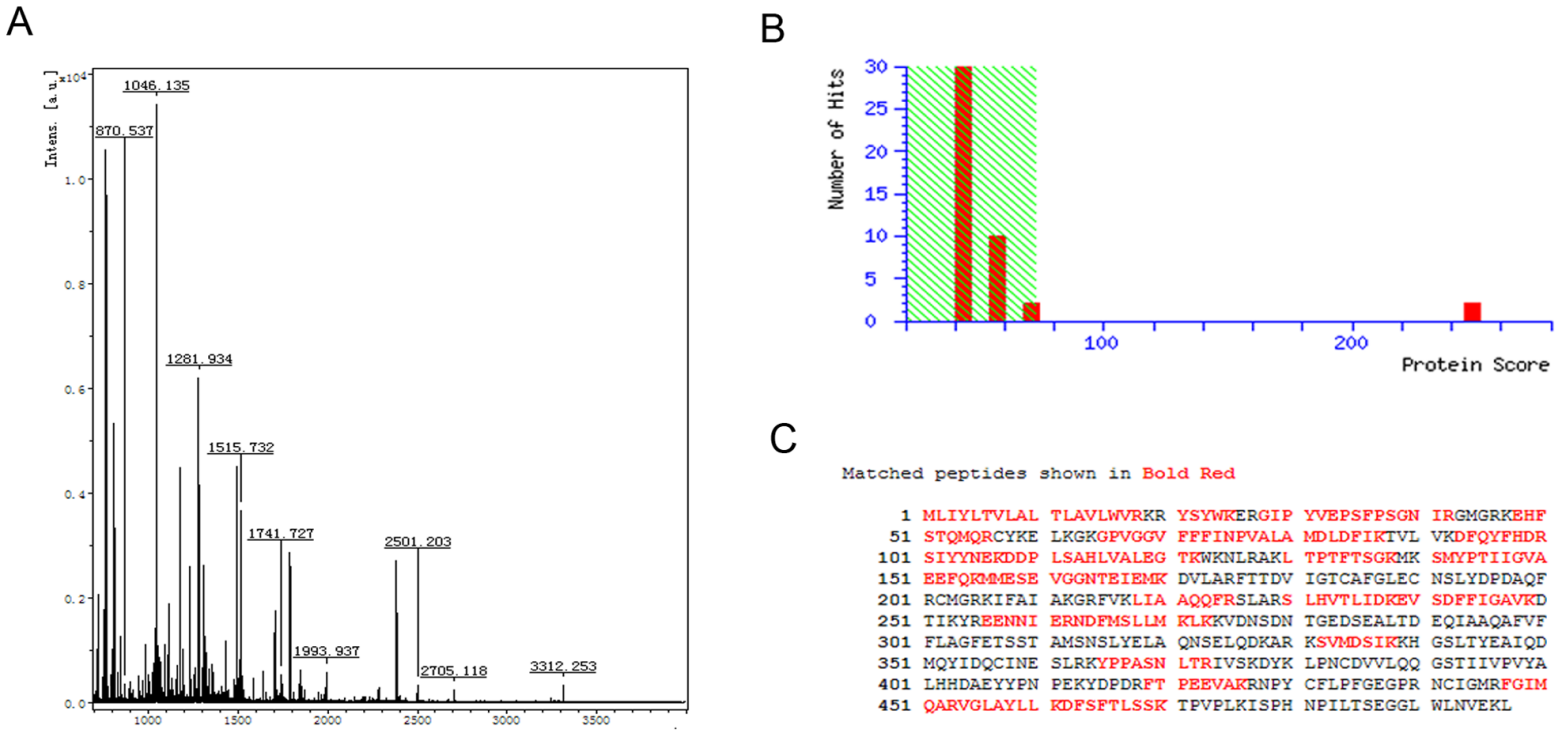
Putative RPS29 interacting proteins identified by NCBInr database searches using peptide sequences from MS/MS. (A) Peptide mass fingerprint spectra. (B) MASCOT scores (*P*<0.05; number of hits = 20). (C) Full-length amino acid sequence of CYP6N3. Red marks indicate peptides identified by MS/MS fingerprinting that match calculated molecular weights.

In order to identify proteins interacting with RPS29, TAP was employed. This technique allows rapid purification under native conditions of complexes expressed at their natural levels. The TAP tag comprises two IgG binding domains consisting of streptavidin and a calmodulin binding peptide (CBP) separated by a TEV protease cleavage site. The N-terminally TAP-tagged RPS29 gene was stably expressed from the pIB-V5-His expression vector in C6/36 cells, as shown by Western blot using anti-His antibody (Fig. 1A). SDS-PAGE and silver staining of TAP assay products revealed discrete protein bands that were removed and analyzed by mass spectrometry (Fig. 1B). Eleven candidate proteins were detected by TAP, and CYP6N3 was the focus of the remainder of this study.Peptide mass fingerprinting (PMF) and NCBInr database searches using peptides sequenced by MS/MS identified CYP6N3, a member of the CYP450 superfamily, as a potential RPS29 interacting protein (Fig 2).

### Confirmation of CYP6N3 binding by GST pull-down, immunofluorescence and confocal laser scanning microscopy and fluorescence resonance energy transfer (FRET)

**Figure 3:**
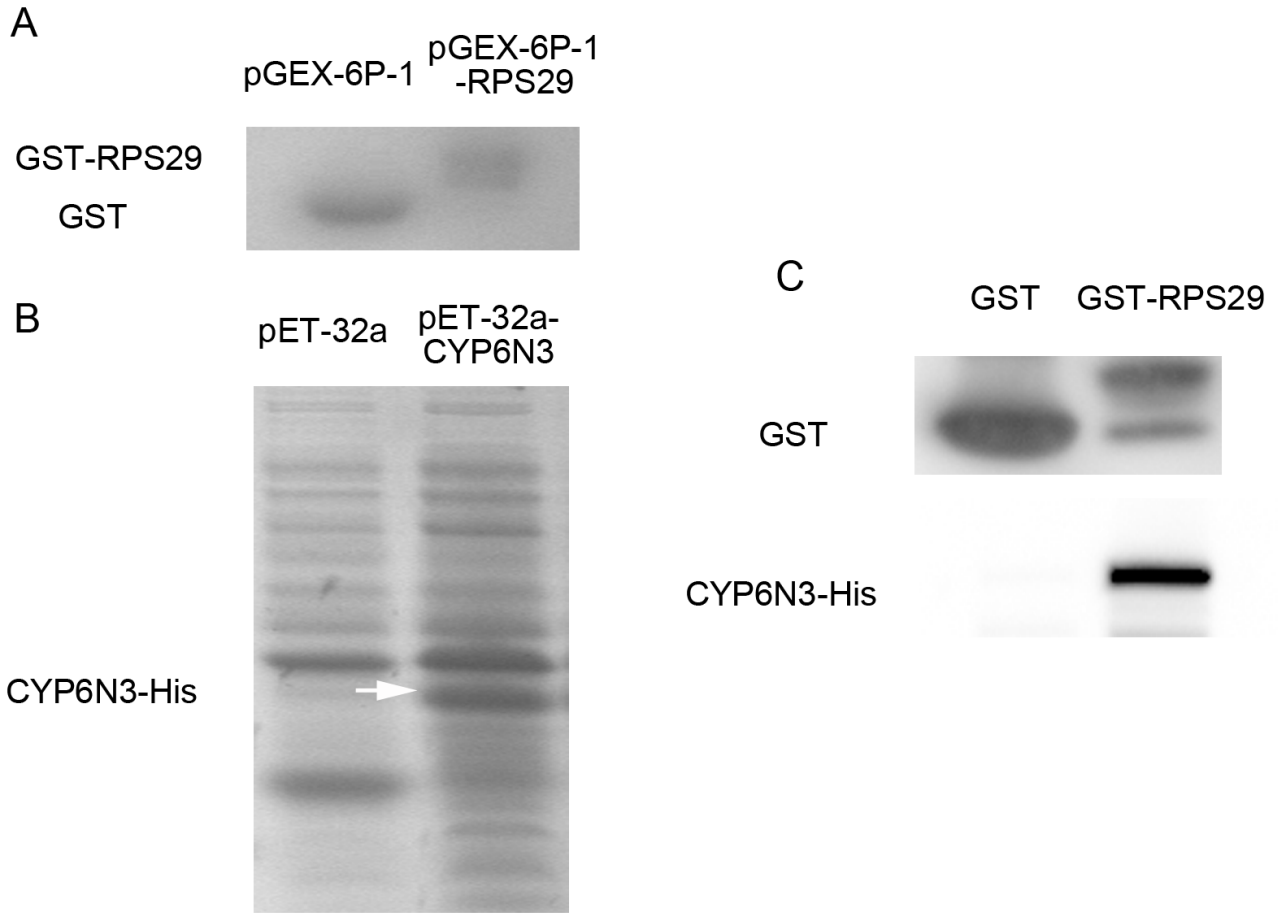
CYP6N3 binding to RPS29 *in vitro*. (A) *In vitro* translated GST and GST-RPS29 visualuzed by commassie blue staining. (B) Total lysates containing *in vitro* translated His-CYP6N3 visualized by commassie blue staining (arrowheads). (C) GST-RPS29 incubated with *in vitro* translated His-CYP6N3 gave a potential complex that was isolated using GST affinity chromatography and detected by Western blotting using anti-His antibody.

Binding between RPS29 and CYP6N3 was confirmed by GST pull-down and immunofluorescence. The cDNAs of RPS29 and CYP6N3 were cloned into the pGEX-6p-1 and pET32a vectors, respectively. *In vitro* translated GST-RPS29 and His-CYP6N3 were identified by commassie blue staining (Fig. 3A and B). GST-RPS29 was immobilized on glutathionine sepharose resin and used to capture CYP6N3 (GST was used as a control). GST-RPS29 but not GST bound to *in vitro* translated CYP6N3 (Fig. 3C). This demonstrated direct binding between RPS29 and CYP6N3 without cofactors.

**Figure 4:**
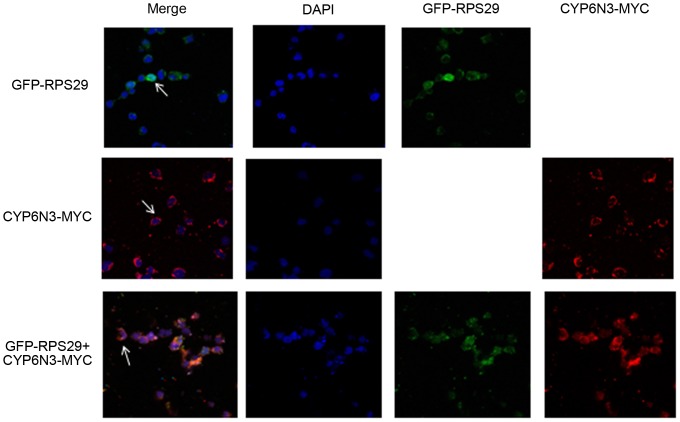
Intracellular co-localization of CYP6N3 and RPS29. C6/36 cells were transfected with GFP-RPS29, CYP6N3-MYC and GFP-RPS29 + CYP6N3-MYC, repectively. Fuorescence was visualized and recorded using confocal laser scanning microscopy after 48 h of expression. CYP6N3 was localized in the cytoplasm (arrowheads) and RPS29 was distributed throughout the cell. RPS29 + CYP6N3 were co-localized in the cytoplasm (arrows). Cells nuceli were stained with DAPI.

To demonstrate binding *in vivo*, immunofluorunsence and confocal laser scanning microscopy were employed. Constructs containing MYC-tagged CYP6N3 and GFP-tagged RPS29 were co-transfected into C6/36 cells. CYP6N3-MYC samples were stained with rhodamine and visualized using a confocal laser scanning microscope. RPS29 fluorescence was distributed throughout the cell, while CYP6N3 fluorescence was localized in the cytoplasm (Fig. 4). The intense fluorescence signal of GFP-tagged RPS29 in the cytoplasm overlapped with the MYC-tagged CYP6N3 signal, indicating an interaction between RPS29 and CYP6N3 *in vivo*.

To further determine that RPS29 could interact with CYP6N3, FRET was used. Constructs containing RFP-tagged CYP6N3 and GFP-tagged RPS29 were co-transfected into C6/36 cells, and the fluorescence were visualized using a FRET microscope (Fig. S1). The results of FRET show that RPS29 could interact with CYP6N3 *in vivo* (Table S1).

### Overexpression and silencing of RPS29 and CYP6N3 in C6/36 cells

**Figure 5:**
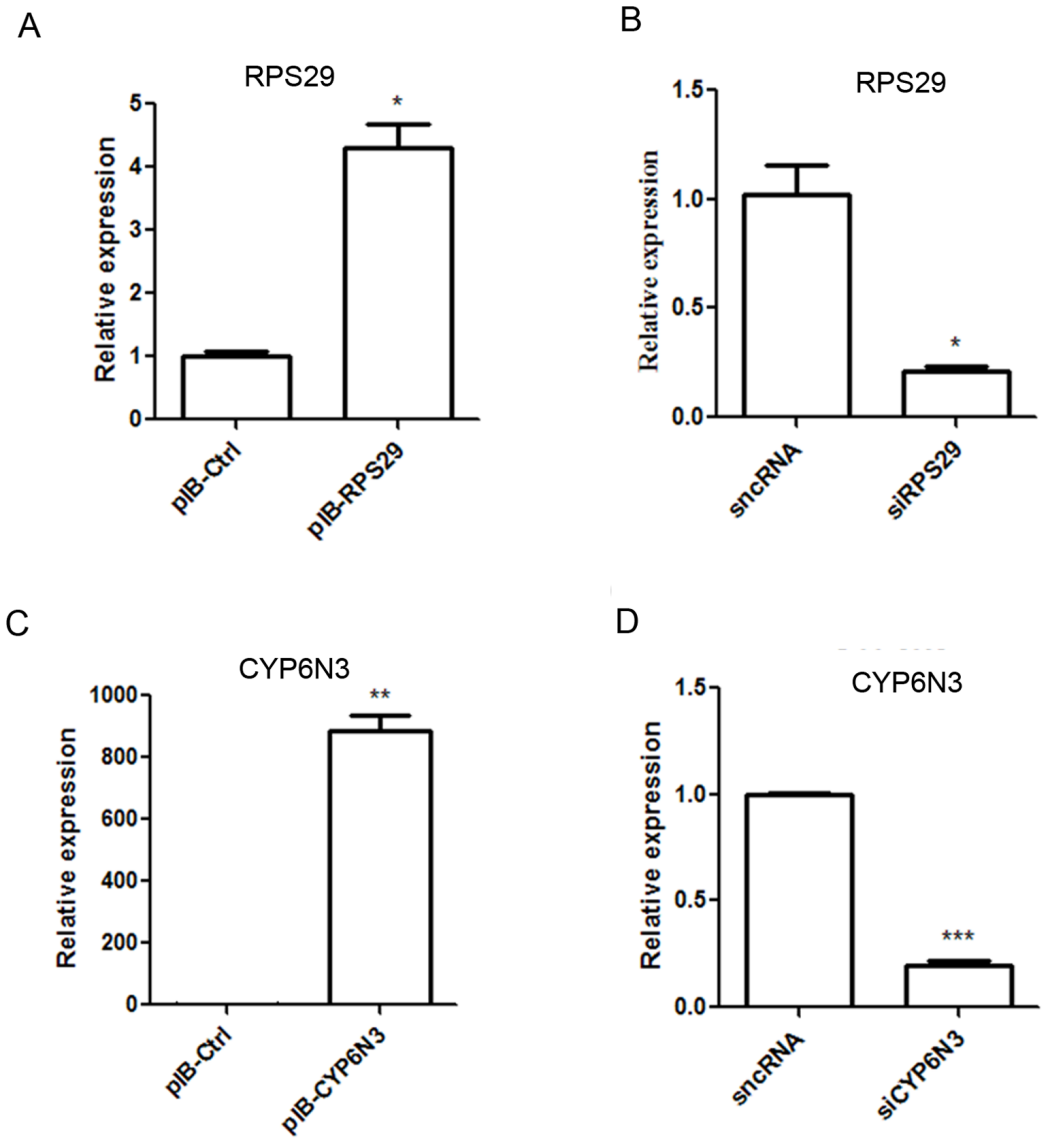
Characterization of transiently transfected cells. Real-time PCR was used to measure RPS29 and CYP6N3 expression following transfection with expression plasmids and siRNAs. Expression was normalized against β-actin.

Real-time PCR was used to assess transfection and expression efficiency of RPS29 and CYP6N3 in C6/36 cells. The mRNA levels of RPS29 and CYP6N3 were significantly increased in cells transfected with pIB-RPS29 and pIB-CYP6N3, respectively (Fig. 5A and B). Cells were transiently transfected with RPS29 and CYP6N3 siRNAs, and expression of both proteins was significantly downregulated compared with the silencer negative control (sncRNA).

### The RPS29-CYP6N3 interaction regulated DM resistance in C6/36 cells

**Figure 6:**
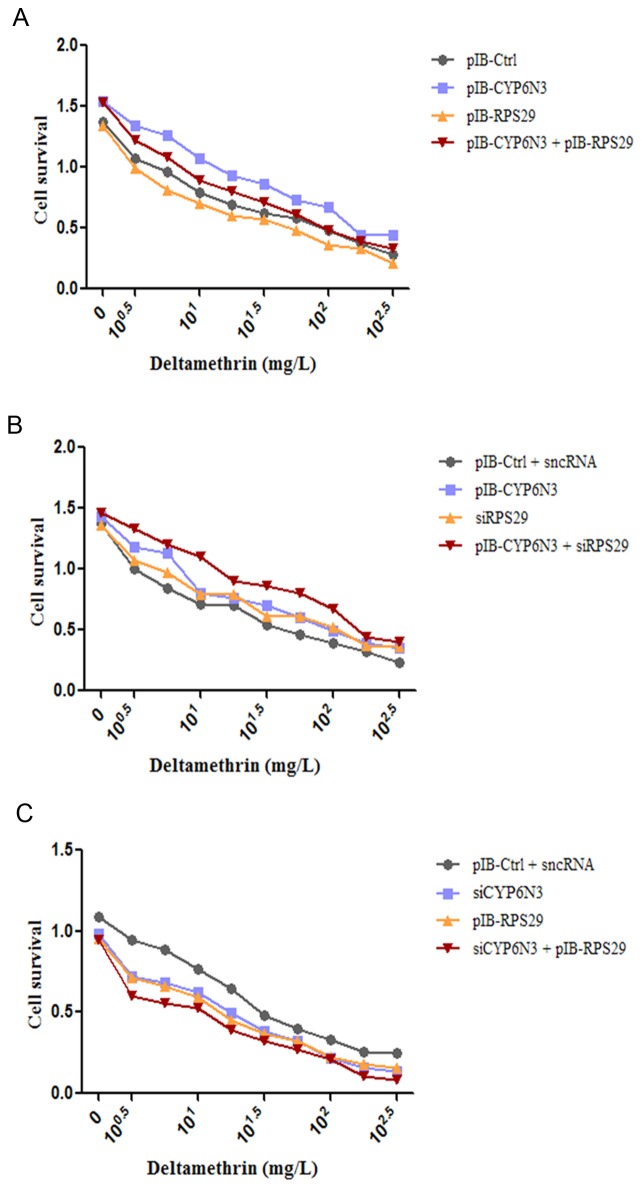
The RPS29-CYP6N3 interaction regulates DM resistance in C6/36 cells. (A) CCK-8 assay of cells transfected with empty vector, pIB-RPS29, pIB-CYP6N3, and pIB-RPS29 + pIB-CYP6N3, in the presence of varying concentrations of DM. (B) CCK-8 assay of cells transfected with empty vector + sncRNA, pIB-CYP6N3, siRPS29, and pIB-CYP6N3 + siRPS29, in the presence of varying concentrations of DM. (C) CCK-8 assay of cells transfected with empty vector + sncRNA, siCYP6N3, pIB-RPS29, and siCYP6N3 + pIB-RPS29, in the presence of varying concentrations of DM.

To investigate the RPS29-CTP6N3 interaction in relation to insecticide resistance, cell viability in the presence of the common insecticide DM was assessed over a wide range of DM concentrations (0–10^2.5^ mg/l) using the CCK-8 assay. Cells transfected with pIB-RPS29 were more susceptible to DM, and RPS29 knockdown significantly enhanced cell viability (Fig 6), as we reported previously. However, CYP6N3 knockdown significantly reduced cell viability, while CYP6N3 overexpression significantly enhanced cell viability. Furthermore, this enhancement was inhibited by RPS29 overexpression and stimulated further by lowered RPS29 expression. Additionally, cell viability in the siCYP6N-transfected cells was lower than the control group and significantly inhibited by RPS29 overexpression. Together, these results indicate that CYP6N3 contributes to enhance the resistance of C6/36 cells to DM, and RPS29 could abrogate the contribution of CYP6N3 to resistance.

### RPS29 promotes proteosomal degradation of CYP6N3

**Figure 7:**
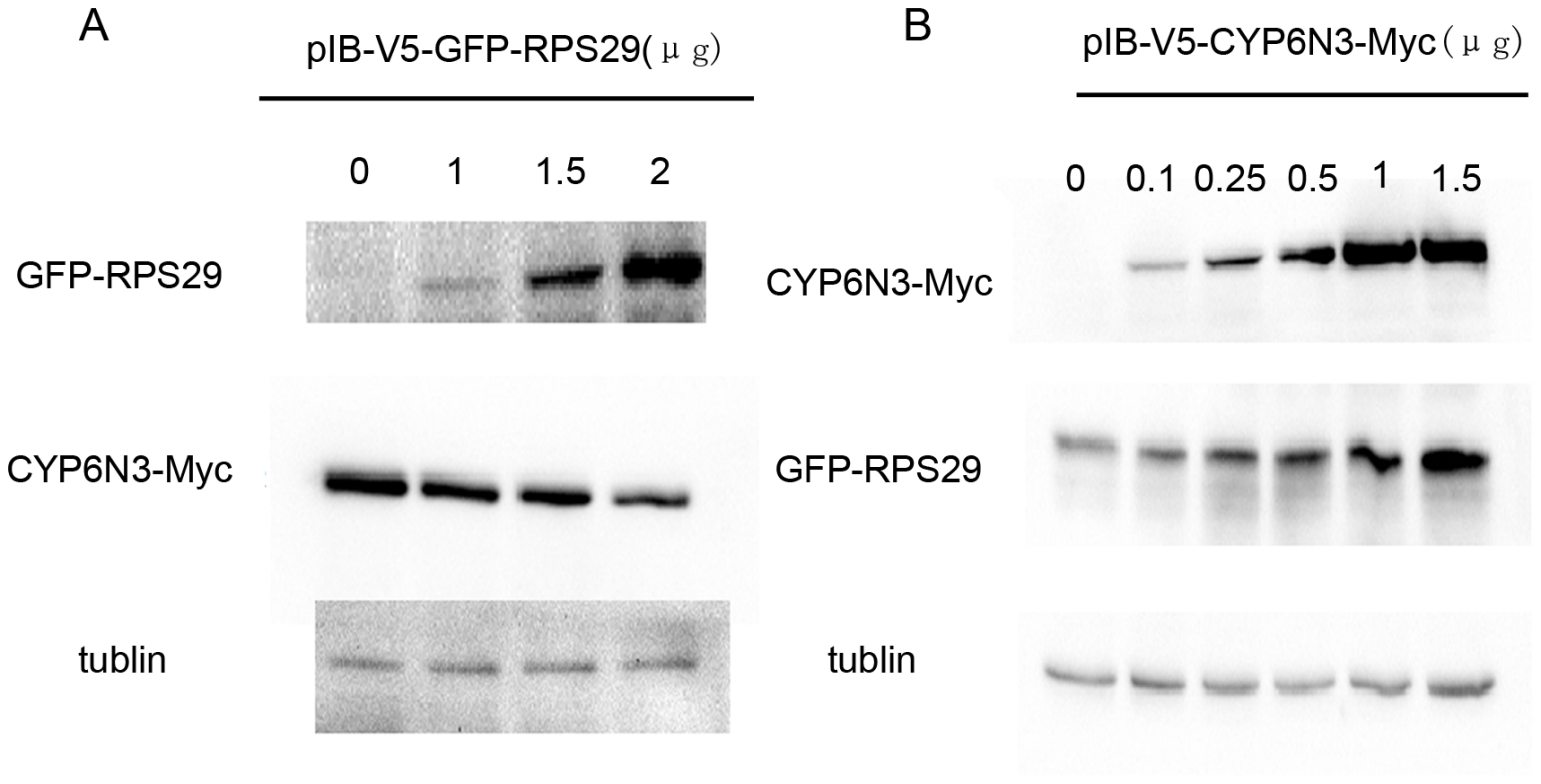
Regulation of CYP6N3 and RPS29 protein levels by RPS29 and CYP6N3 overexpression. (A) Co-transfected C6/36 cells expressing varying amounts of GFP-RPS29 and a constant amount of CYP6N3-MYC (empty vector + CYP6N3-MYC was used as a control). CYP6N3 was detected by Western blotting using anti-MYC antibody 24 h after transfection. (B) Co-transfected C6/36 cells expressing varying amounts of CYP6N3-MYC and a constant amount of GFP-RPS29 (empty vector + GFP-RPS29 was used as a control). RPS29 was detected by western blotting using anti-GFP antibody 24 h after transfection.

**Figure 8:**
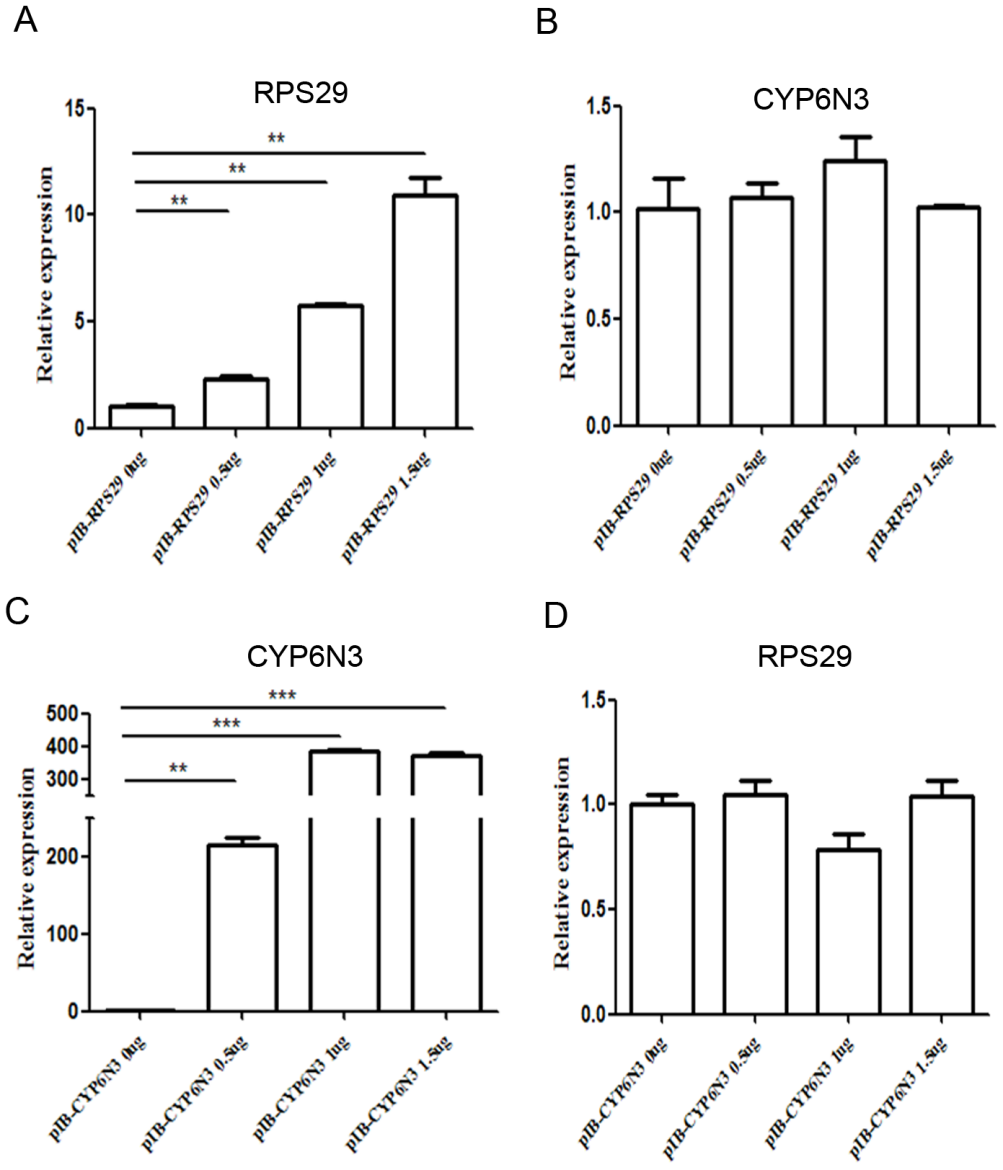
Real-time PCR showing mRNA levels of CYP6N3 and RPS29 are comparable to RPS29 or CYP6N3 transfected cells. (A) Transfected C6/36 cells expressing varying amounts of RPS29 (empty vector was used as a control). Real-time PCR of CYP6N3 mRNA levels 24 h after transfection. (B) Transfected C6/36 cells expressing varying amounts of CYP6N3 (empty vector was used as a control). Real-time PCR of RPS29 mRNA levels 24 h after transfection. Expression was normalized against β-actin.

**Figure 9:**
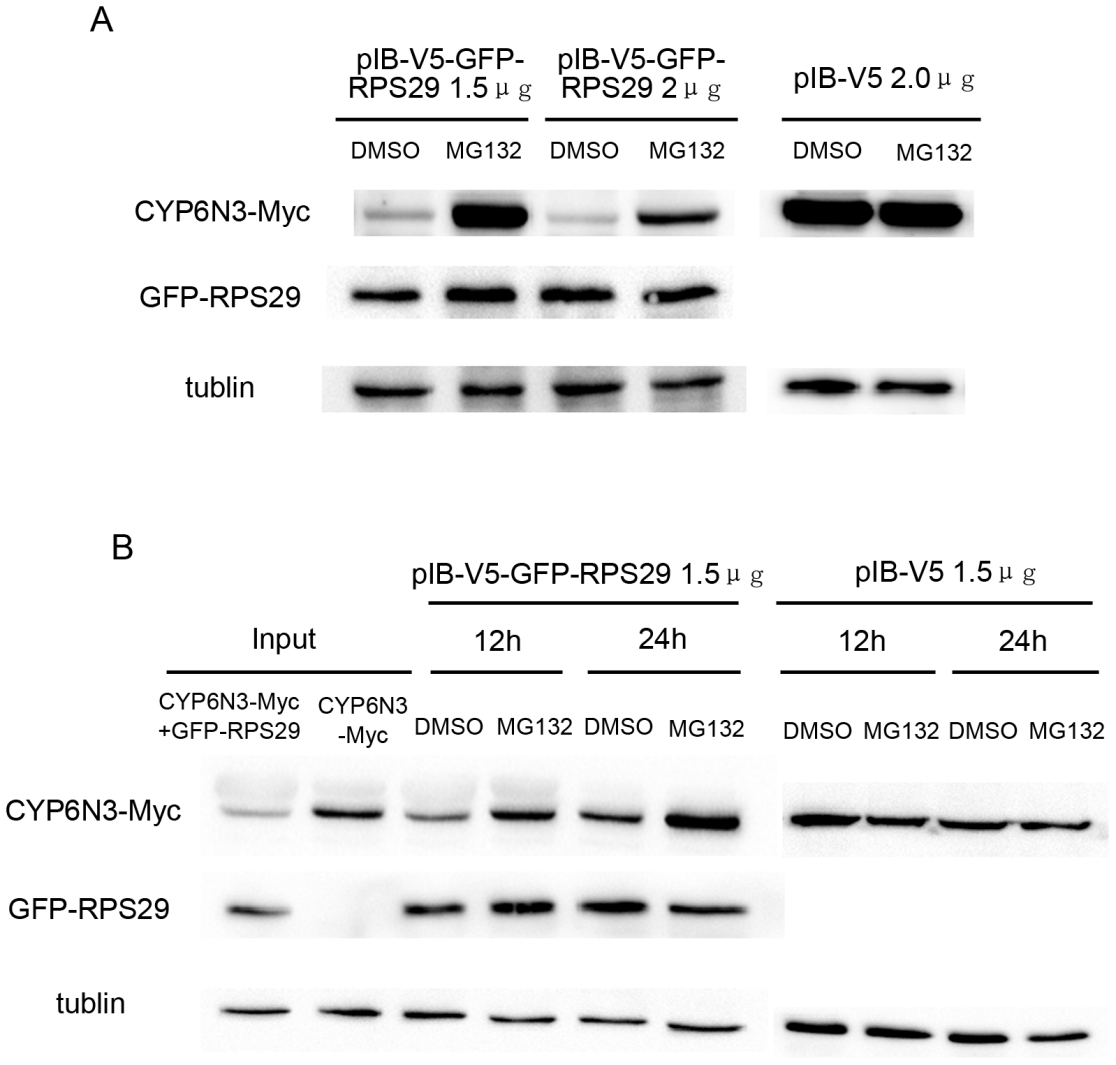
Expression of CYP6N3 is regulated by proteasome-mediated degradation. (A) Transfected C6/36 cells expressing varying amounts of GFP-RPS29 were exposed to the protesome inhibitor MG132 (20 µM). Empty vector was used as a control. (B) C6/36 cells were transfected with GFP-RPS29 or control vectors and exposed to the protesome inhibitor MG132 (20 µM) for different time periods.

To further investigate the RPS29-CYP6N3 interaction, cells were transiently co-transfected with constructs encoding GFP-RPS29 and CYP6N3-MYC (Cells transfected with CYP6N3-Myc and empty vector were used as a control). Overexpression of RPS29 resulted in a dose-dependent decrease in CYP6N3 protein levels (Fig. 7A). However, RPS29 expression was increased when CYP6N3 was overexpressed (Fig. 7B). This alteration in CYP6N3 and RPS29 levels was likely due to post-translational regulation because the mRNA levels of both CYP6N3 and RPS29 were comparable to those of RPS29 or CYP6N3 transfected cells as determined by real-time PCR experiments (Fig. 8). To determine whether the low protein level of CYP6N3 was indeed due to decreased protein stability, cells were treated with MG132, a specific inhibitor of proteasomes. CYP6N3 protein levels were clearly recovered in the MG132-treated cells expressing different amounts of RPS29, and the recovery was time-dependent (Fig. 9).

## Discussion

Ribosomal protein RPS29 was found to be associated with DM resistance in our previous study [14]. To investigate the mechanism, TAP was used to identify RPS29-interacting paterners. The TAP tag has been specifically designed to maintain expression of the fusion protein at its natural level, and the method allows rapid purification under native conditions. Purified complexes can also be subjected to structural and functional analyses [17], [18]. CYP6N3 was identified as a protein capable of binding to RPS29 protein by the TAP method, and this putative interaction was confirmed using a further two complementary approaches. GST pull-down assays demonstrated direct binding between RPS29 and CYP6N3 *in vitro*, and immunofluorescence combined with confocal microscopy and FRET verified interaction of CYP6N3 and RPS29 *in vivo*. This study is the first to report such a finding.

Overexpression of RPS29 in C6/36 cells lowered CYP6N3 protein levels in a dose-dependent manner, while mRNA levels were unchanged. Furthermore, overexpression of CYP6N3 resulted in an increase in RPS29 protein levels. Together, these results suggested that the reduction of CYP6N3 is due to post-translational regulation by RPS29, rather than competitive inhibition of expression. Post-translational regulation can take many forms. In this study, CYP6N3 appeared to be degradaded after interacting with RPS29. In eukaryotic cells, degradation of cellular proteins is performed primarily by the proteasome system and autophagy [19]. We treated the cells with MG132, a specific inhibitor of the proteasome, and observed accumulation of CYP6N3, strongly suggesting proteosomal degradation of CYP6N3. The 26S proteasome is a large multiprotein complex comprised of a 20S proteolytic subunit that is capped at both ends by 19S regulatory subunits [20], [21]. Recent studies revealed that the core catalytic complex of the eukaryotic 20S proteasome has at least five different peptidase activities, and the functions of the 19S regulatory complex are recognition of the polyubiquitinated substrate, release of the polyubiquitin chain, and translocation of the unfolded substrate towards the catalytic sites of the 20S proteasome [22], [23]. In eukaryotes, 26S proteasomes degrade ubiquitinated and non-ubiquitinated proteins in an ATP-dependent manner [24], [25]. In this study, CYP6N3 was degraded by the proteasome when interacting with RPS29. Whether or not this is dependent on ubiquitination needs further investigation.

Insecticide resistance has limited the effective control of populations of insect pests and presents an obstacle to the control of vector-borne diseases across the world [8]. Understanding the molecular mechanisms of insecticide resistance is essential for effective monitoring and control of insect vectors. Metabolic resistance involves alterations in the expression of enzymes and detoxification pathways, and is probably the most ubiquitous resistance mechanism, although target resistance is also very common. The mechanisms of metabolic resistance are not well understood, but often occur through enhanced biodegradation of the insecticide, usually via overexpression and/or elevated activity of three major enzyme families; CYP450s, esterases, and glutathione S-transferases [26], [27], [28]. Of these, CYP450 enzymes bind and/or metabolize pyrethroids, and are the primary enzyme family associated with resistance to many insecticides including pyrethroids such as DM [28], [29]. CYP450s such as CYP6A1, CYP6D1, CYP6P3, and CYP6M6 have been associated with insecticide resistance in insects [30], [31], [32], [33]. CYP6N3 is a member of the CYP 6 class of CYP450s. Overexpression of CYP6N3 stimulated resistance to DM in this study, suggesting this CYP450 enzyme is also involved in metabolic insecticide resistance. RPS29 overexpression diminished cell viability while CYP6N3 overexpression enhanced cell viability, and this enhancement was inhibited by RPS29 overexpression. Together, these results suggested that RPS29 abrogated the CYP6N3-associated resistance in C6/36 cells by binding to and targetting CYP6N3 for proteosomal degradation.

## Supporting Information

Figure S1 **Three channel images of GFR-RPS29 and CYP6N3-RFP.** C6/36 cells were transfected with GFP-RPS29, CYP6N3-RFP and GFP-RPS29 + CYP6N3-RFP, repectively. Fuorescence was visualized and recorded using a FRET microscopy after 48 h of expression.
(TIF)

Table S1 **In vivo interaction between RPS29 and CYP6N3 detected by FRET.**
(DOCX)
