# Supplementary figures and images for: Ribosomal Protein S29 Regulates Metabolic Insecticide Resistance through Binding and Degradation of CYP6N3
Source: PLoS One. 2014 Apr 11;9(4):e94611. doi: 10.1371/journal.pone.0094611 (PMC3984272; doi:10.1371/journal.pone.0094611)

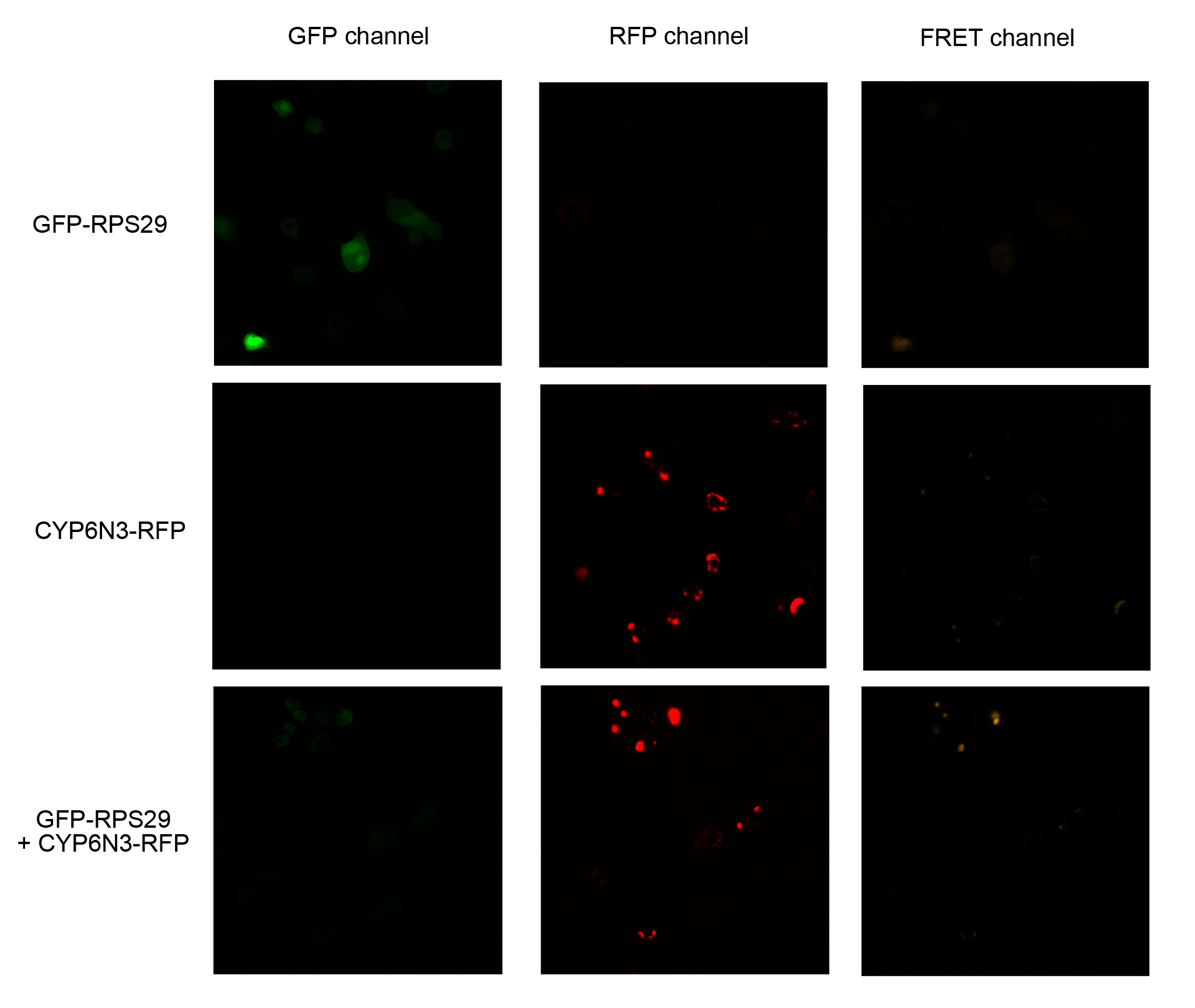

Supplement: Figure S1 — Three channel images of GFR-RPS29 and CYP6N3-RFP. C6/36 cells were transfected with GFP-RPS29, CYP6N3-RFP and GFP-RPS29 + CYP6N3-RFP, repectively. Fuorescence was visualized and recorded using a FRET microscopy after 48 h of expression. (TIF) [file pone.0094611.s001.tif]
