# Supplementary material for: Ribosomal Protein S29 Regulates Metabolic Insecticide Resistance through Binding and Degradation of CYP6N3
Source: PLoS One. 2014 Apr 11;9(4):e94611. doi: 10.1371/journal.pone.0094611 (PMC3984272; doi:10.1371/journal.pone.0094611)
Supplement: Table S1 — In vivo interaction between RPS29 and CYP6N3 detected by FRET. (DOCX) [file pone.0094611.s002.docx]

**Table S1. In vivo interaction between RPS29 and CYP6N3 detected by FRET**

|  | The fluorescence intensity of FRET (F_f_) | The fluorescence intensity of GFP (D_f_) | The fluorescence intensity of RFP(A_f_) | Parameter a | Parameter b | F^c^ |
| --- | --- | --- | --- | --- | --- | --- |
| pIB-V5-GFP | 29.41 | 76.33 | _ | _ | 0.39 | _ |
| pIB-V5-RFP | 12.489 | _ | 36.46 | 0.34 | _ | _ |
| pIB-V5-GFP-RPS29 | 21.75 | 40.54 | 14.13 | _ | - | 1.14 |
| pIB-V5-CYP6N3-RFP | 26.75 | 5 | 72.05 | _ | _ | 0.3 |
| pIB-V5-GFP-RPS29  + pIB-V5-CYP6N3-RFP | 35.67 | 17.48 | 56 | _ | _ | 9.81 |

Note: 1. F^c^ stands for the fluorescence intensity for fluorescence resonance energy transfer. F^c^ = F_f_ - a * A_f_ – b * D_f_

2. All values ​​mean the average of three results.
